# An Ecological Accounting System for Integrated Aquatic Planning and Habitat Banking with Case Study on the Toronto Waterfront, Ontario, Canada

**DOI:** 10.1007/s00267-021-01531-5

**Published:** 2022-02-02

**Authors:** Susan E. Doka, Charles K. Minns, Brent G. Valere, Steven J. Cooke, Rick J. Portiss, Thomas F. Sciscione, Alwyn Rose

**Affiliations:** 1 Fisheries and Oceans Canada, Great Lakes Laboratory for Fisheries and Aquatic Sciences, 867 Lakeshore Rd., Burlington, ON L7S 1A1 Canada; 2 Department of Ecology and Evolutionary Biology, University of Toronto, 25 Willcocks Street, Toronto, ON M5S 3B2 Canada; 3 Fisheries & Oceans Canada, Fish and Fish Habitat Protection Program, 867 Lakeshore Rd., Burlington, ON L7S 1A1 Canada; 4 Fish Ecology and Conservation Physiology Laboratory, Department of Biology, Carleton University, 1125 Colonel By Dr., Ottawa, ON K1S 5B6 Canada; 5 Toronto and Region Conservation Authority, 5 Shoreham Drive, Downsview, ON M3N 1S4 Canada; 6 Fisheries & Oceans Canada, Ecosystems Management Policies and Practices, 200 Kent Street, Ottawa, ON K1A 0E6 Canada; 7 Environment and Climate Change Canada, Canadian Wildlife Service, Place Vincent Massey, 351, boul. Saint-Joseph (14th Floor), Gatineau, QC K1A 0H3 Canada

**Keywords:** Ecological accounting, Fish habitat, Integrated planning, Natural reserves, Offsetting, Habitat restoration

## Abstract

A key aspect of contemporary fish habitat management is the need to account for losses and gains associated with development and offsetting measures while protecting high quality features. We propose an ecological accounting framework for aquatic ecosystems using habitat equivalents scaled to aquatic productivity, and using fish-to-habitat associations by life stage, based on local fish community needs. The framework uses both landscape-scale and site-level evaluations of pre- and post-project habitat changes to assign and track habitat parcels, using ecological baselines and fish-habitat target setting. Concepts of natural capital reserves and productivity-based ecotypes are used for trading losses and gains between impacts from development projects and offsets, including restoration actions, while maintaining ecologically important areas intact. Traditional accounting terms such as deposits, withdrawals, and transfers are defined using scaled habitat-equivalents as the currency. Other key features of the framework include setting a service area that is ecologically meaningful, and conducting habitat transactions guided by habitat conservation, protection, and restoration (*habitat CPR*) principles. The nearshore area of the Toronto and Region is used as a case study to illustrate the eco-accounting framework and how habitat banking could be incorporated along with planned restoration to remediate this degraded but continually developed area. The framework represents significant advances in managing cumulative habitat effects in an integrated way, moving away from a focus on only project- or site-level assessments. We feel this approach could be adapted to other ecosystem types in addition to the lake, nearshore area example provided here.

## Introduction

In Canada, evaluations of projects in or near water have typically focused on site-level, one-time fish habitat evaluation and assessment when offsetting (i.e. trading unavoidable adverse effects for offsetting measures) on a project-by-project basis. What is needed is an environmental planning process to set habitat trades within an integrated aquatic landscape. An ecological accounting framework could be used to weigh decisions about unavoidable impacts from new or proposed development within integrated conservation, protection, and restoration planning (habitat *CPR plans*), and to calculate habitat equivalency trades within a landscape-scale, *service area*. This approach would incorporate habitat offsetting into the overall integrated spatial planning and management of aquatic ecosystems. Elements of an aquatic, ecological accounting framework have been used individually, though have rarely been unified or integrated as we suggest.

**Fig. 1:**
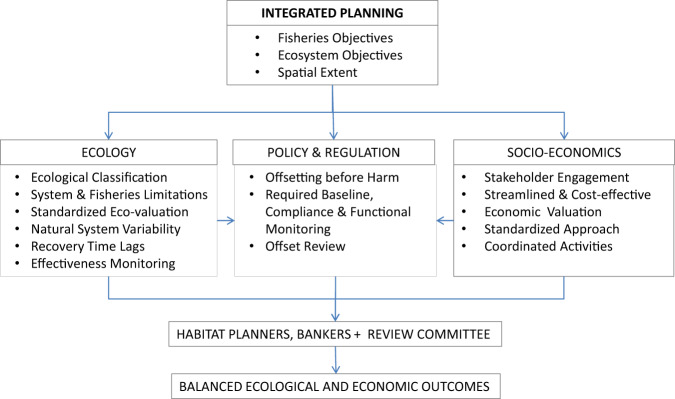
Flow diagram of how ecological, regulatory, and economic components could be customized for a Toronto Region nearshore integrated planning area. Connections between area management strategies (e.g., the TWAHRS) and relevant conservation objectives (e.g., Lake Ontario Fisheries Management Objectives (Great Lakes Fishery Commission; Stewart et al. 2017), Lake Ontario Binational Biodiversity Conservation Strategy Working Group (LOBSWG 2009)), government agencies (both science and regulatory), and habitat bankers (e.g., TRCA advised through Aquatic Habitat Toronto), and proponents and stakeholders (e.g., Waterfront Toronto, public, First Nations) are highlighted. Bullets highlight the contributions from ecology, policy, and regulatory frameworks, and benefits for integrated management and socio-economics

Habitat banking approaches (McKenney and Kiesecker 2010; Hunt et al. 2011; Noga and Adamowicz 2014) are known by a wide range of other names, including conservation offset programs, biodiversity banking or offsets, market-based conservation, mitigation banking, and ecological terrestrial or aquatic planning (IUCN 2016; zu Ermgassen et al. 2019). To date, approaches have mainly been used for terrestrial, habitat trading and wetlands conservation (Haines-Young 1999; Loughlin and Clarke 2014). Further, few habitat banking studies have explicitly described how ecological accounting or equivalency trading is conducted over the longer-term in a consistent manner within a landscape context. Notable exceptions include Haines-Young (1999), who discussed how changes in land cover accounts in the U.K. might be incorporated into national state-of-the-environment accounts (Goddard et al. 2009). In aquatic ecosystems, habitat banking refers to the concept of trading habitat equivalents among parcels of the aquatic landscape to balance conservation objectives with development approvals, where the compensation project or offset is built prior to an impact (DFO Fisheries and Oceans Canada (2013), DFO Fisheries and Oceans Canada (2019)), preferably within the same landscape-scale service area. An ecological habitat accounting system is needed for integrated planning, one that may link to social, institutional, and financial goals that are also being considered and reconciled (McKenney and Kiesecker 2010). Here, we consider only the ecological connections in the framework but the broader connections are shown in Fig. 1.

Good accounting practices are central to the successful operation of financial institutions. The same is true for implementing integrated planning for aquatic habitat CPR. Practically, there are many useful analogies between commercial and ecological banking systems, and terms such as *deposits*, *withdrawals*, and *transfers* can also be applied within an ecological accounting framework. A habitat service area contains ecosystem components (*natural capital*), that may be valuated through standard ecological measures (*habitat equivalencies*), and could include deposits from natural capital generation, such as gains from habitat restoration projects. An integrated management plan may identify particular conservation projects (banked or true deposits) that are needed within a service area where future development projects or other adverse activities (withdrawals) are anticipated, but also can exceed equivalency if the current situation is degraded.

The banking area may also already contain or generate natural *capital reserves*; areas with significant ecological importance or productivity that are set aside to be protected and are not available for trading. Recent investigations for bird communities recommend that where possible, up to 50 to 85% of managed areas should remain natural to preserve their ecological functionality (Ford et al. 2009). The United Nations recommends 50% (half of Earth) be protected with an interim target of 30% protected by 2020, especially freshwaters and wetlands (UN United Nations (2021)). Currently the target in Canada is 17% of land and water by 2023 (which includes restoration of degraded areas, such as Toronto’s waterfront.

Integrated management of habitat infers that future withdrawals are balanced with existing and future deposits towards conservation or net-gain goals. Depending on the currency or equivalency unit chosen, the offsetting needed to counter adverse impacts from a proposed project may be geographically removed from the project site, but be in-line with the desired outcomes of fisheries management goals within the overall integrated service area. Rarely, depending on circumstances and with additional justification, a transfer of gains and losses between different service areas could be considered to help offset a withdrawal if sufficient habitat deposits cannot be implemented within the impacted service area.

Different types of ecological currencies can be used for trading withdrawals for deposits, and these are reviewed in Clarke and Bradford (2014) for fisheries or habitat management. As with other approaches to valuing ecosystem goods and services, the units of valuation (the common currency or habitat equivalency) for the traded habitat parcels depends on the habitat bank’s primary purpose for accounting. For example: carbon dioxide equivalents are used to mitigate climate change impacts (Bishop and Hill 2014); habitat-based areas or suitabilities are used in conservation planning for fishes or birds (Marxan: Airamé et al. 2003, Game and Grantham 2008; HAAT: Minns et al. 2001); and the relative productivity of individual species, or communities in a multi-species fishery, has been proposed (Randall et al. 2013) as well as standardizing across life stages of a species by using adult equivalents per area (Bradford et al. 2015). Valuation can involve both ecological and socioeconomic factors if desired; however, care must be taken with additionality, such that distinct ecosystem services or benefits gained (e.g., carbon credits, habitat supply gains, wetland area targets) are bundled as one offset trade and not stacked, or used as separate credits in different trades (Cooley and Olander 2011; Gillenwater 2012).

### Establishing an Ecological Accounting Framework

We propose that ecological accounting for integrated planning and aquatic habitat banking involve ecological valuation and mapping of a baseline or reference condition (if needed, or available), but at minimum, current conditions. Subsequently, valuation and tracking of all habitat transactions happens within an integrated planning or service area. Typical currencies for valuation of aquatic habitat centre around fish diversity or fisheries productivity surrogates. Moving from habitat features to productivity measures involves more uncertainty in the equivalency calculations (Bradford et al. 2016). We propose quality-based habitat metrics, with a scalar to account for varying productivities, as the currency for equivalency calculations for fish habitat trading.

Quality-based equivalencies rely on habitat associations or niche information for single species, guilds, or whole fish communities, and can be based on expert opinion or quantitative approaches. Examples include habitat assessment tools like the Habitat Ecosystem Assessment Tool (HEAT: Minns et al. 2001; Abdel-Fattah et al. 2021), Marxan (Airamé et al. 2003; Game and Grantham 2008), and suitability-based approaches (Zorn et al. 2011; many are reviewed by de Kerckhove et al. [2008]). The tools are used to evaluate the relative habitat quality and supply across different habitat parcels. A key assumption of the approaches is that relative habitat suitability or quality assignments (albeit based on the ecological needs of the organisms using those habitats) can be a surrogate for relative productivity differences (Randall and Minns 2002; Randall et al. 2012) if not directly measured. While the output values may reflect the relative importance of different habitats for different processes (if multiple life stage requirements are assessed), the suitability values do not necessarily scale properly to the absolute differences in production potential between mesoscale habitats (such as wetlands, embayments in lakes, or open coast), herein referred to as major *ecotypes*. This represents a challenge for area-based management and habitat accounting; a standardized currency and exchange rates for trading between ecotypes are required. We propose maximum potential production or maximum potential productivity be used, if we assume resource production or ecological productivity can be scaled from habitat values (i.e., relative fisheries production can be roughly estimated from the relative quality or usability of microscale habitat features within distinct ecotypes).

Aside from a common currency, basic conservation planning tenets need to be followed when establishing and operating a landscape-scale habitat accounting system (Lindenmayer and Hunter 2010; Linke et al. 2010). Early considerations and decisions in establishing and operating such an accounting system include:

- synthesizing *ecosystem goals* from applicable management plans for a locale, which could inform

- the *geographic bounds* of the habitat bank service area or integrated planning area, within which the habitat transactions will take place;
- defining the *ecotypes* within the area bounds, and what currency (*habitat equivalency*) is to be used;
- determining *time lags* needed for an banked offset or restoration project to attain the full ecological value or expected productivity, and how this relates to trade timing, and;
- identifying *important existing natural features* (remaining or previously restored) that should be set aside and conserved.

These early fundamentals should be based on regional empirical data, other scientific evidence or knowledge bases, and established at the outset of planning. In subsequent sections, we expand on each fundamental and focus on the functional operation of a habitat accounting scheme. The purposes of which are: reconciling impacts, habitat banks or offsets, and restoration projects; maintaining ecological functions for fishes; linking that functionality to productivity while still recognizing the fish community’s need of diverse habitats; and operationalizing fisheries objectives (e.g., Stewart et al. 2013) for an area into actionable context. We illustrate—with examples from an ongoing case study along the Toronto region’s waterfront—how the various components of a fish habitat accounting scheme may work (Fig. 1). The immediate goal is to facilitate and track the ongoing operation of the Toronto regional, nearshore integrated planning area, which helps define a service area for habitat banking and other actions. The Toronto regional nearshore case study is briefly described here.

#### Case Study: TWAHRS

In 2003, the Toronto Waterfront Aquatic Habitat Restoration Strategy (TWAHRS: TRCA Toronto and Region Conservation Authority (2003), Prime et al. 2013) was created in anticipation of the development of the Toronto Waterfront Revitalization Initiative (now called Waterfront Toronto) and other ongoing nearshore development in this large urban centre. TWAHRS was developed in response to existing and proposed, large-scale adverse effects involving the destruction and subsequent improvement of fish habitat through coordinated restoration and offsetting practices. The strategy stemmed from the need to create and restore fish and wildlife habitat in a useful way, in keeping with Toronto’s Remedial Action Plan under the *Great Lakes Water Quality Agreement* (GLWQA 1987, 2013). The TWAHRS not only addressed anticipated changes over a 25-year period, but also restoration actions in response to multiple, cumulative stressors that had occurred over a long historical period. A local advisory group, Aquatic Habitat Toronto (AHT), was established with the participation of resource management agencies and the development industry (mainly Waterfront Toronto and the City of Toronto as partners) to implement the Strategy. In 2012, AHT initiated the planning and implementation of a habitat banking strategy as part of proactive offsetting measures to account for ecological time lags involved in restoration and offsetting, and in anticipation of large impacts that couched within an integrated spatial plan (Prime et al. 2013). The accounting methods proposed here stem from this process.

## Methods

Our proposed ecological accounting system for integrated fish habitat CPR planning has several ordered components already outlined. The ecological accounting framework and subsequent *habitat transactions* can only be implemented once these prerequisite conditions are met.

The first condition is identifying or establishing an *ecological strategy* with supporting, evidence-based goals and performance targets, such as local fish community and habitat goals, for the area. Clear SMART (specific, measurable, achievable, realistic, timely; sensu Doran 1981) performance targets are needed to guide the integrated activities, including banking. These targets need to be thorough and ecologically meaningful locally, but also adaptive to allow iterative reviews and updates of: those specific performance targets; the criteria for meeting those targets; and the methods for assessing success in achieving those targets.

Next, and related to the ecological strategy, the *geographic boundaries of the habitat bank service area (or integrated planning area)*, within which the banking or trading will be conducted, need to be defined. This area should be ecologically meaningful to the majority of the species or habitats that local management and plans are meant to conserve, protect, or restore (i.e., *CPR plans*). Fixed planning boundaries and standardized regulations for banking ensure that both the benefits and costs (i.e., environmental, social, and economic) are shared within the same spatial context. Some exceptions (e.g., inter-service area transfers) may be necessary if offset or restoration options within a geographic area are limited. However, those out-of-area trades should be guided by consistently applied rules and transparency in decision-making.

Within the established boundaries, there will typically be recognizably discrete habitat areas belonging to distinct *ecotypes*. These ecotypes, which often differ in their productivity and support different ecological communities, need to be well-defined. Measurable differences in ecotypes are needed to scale or calculate the equivalence of habitat parcels within and between ecotypes for accounting trades of habitat deposits and withdrawals. Therefore, *a methodology for calculating equivalencies* of habitat parcels within ecotypes is also required.

A further consideration is that the integrated planning/service area may already include parcels of high-quality aquatic habitat (natural or previously restored) across the ecotypes present. If there is agreement, those areas should be designated as protected, *natural capital reserves*, ideally based on quantitative evidence or expert local opinion. Also, it is well-documented that restoring lost ecological features does not return their equal original value (Maron et al. 2012, UN 2021), so it is important to identify natural capital reserves before habitat trades begin. Reserves of high-quality natural aquatic habitats must be retained and protected for the long-term resilience of the aquatic resources in an area, especially in degraded systems. In the accounting framework, restored areas are also protected from further trading, especially if required to reach local ecological targets. The parcels/ecotypes and their equivalencies are accounted on balance sheets within the integrated plan area, including natural capital reserves (which can subsequently be added to with continued offsets and restoration projects. Broader scale improvements, like water quality, would improve the baseline or current status in general in the accounts.

### Ecological Strategy: Fish Community and Habitat Goals

Setting goals for biological or fish production, productivity, diversity, or functional use of new or restored habitats, whether for planned restoration or offsetting purposes, is essential. Of equal importance is establishing targets related to the goals in order to measure success. Ecosystem goals and targets from local management plans are a good place to begin to synthesize. For the Toronto nearshore planning area, the TWAHRS includes fishery, habitat, and ecosystem goals (TRCA Toronto and Region Conservation Authority (2003)) as well as local socio-economic and remedial action plan objectives (e.g., improved water quality). However, the set of targets needs to be internally consistent, specific, and quantifiable as well as operationally achievable within realistic timeframes. Hence, periodic re-assessment and review of outcomes will be necessary as baseline conditions may change (e.g., climate change). An integrated planning area’s environmental, land-use, and development history is important to consider as context for setting baseline/benchmark, or performance targets against which CPR plans would be directed. Certain areas could focus on one of conservation (no net loss), protection (reduction of loss), or restoration (net gain), depending on the area’s needs.

In the Toronto region, the loss of 486 hectares (almost 5 km^2^) of wetland, mainly in the Ashbridges Marsh (RCFTW 1991a) at the historic mouth of the Don River, occurred early in the 20th century from rapid urban development that dramatically reduced the local aquatic productivity for desirable aquatic species and their diversity. This productive wetland and barrier-beach complex once supported a diverse fish community and was a primary reason for early settlement. Recreational and commercial fisheries such as Largemouth Bass, Smallmouth Bass, Muskellunge, Northern Pike, Yellow Perch, Pumpkinseed, and Walleye have been affected (Whillans 1979). All these fishes, with the possible exception of Muskellunge, are still present in moderate numbers in the Toronto region of Lake Ontario, but at much lower abundances than historically (Whillans 1996). A goal through habitat restoration in this area is an increase in abundance of these fishes to an average abundance higher than current. However, abundance below historical levels is still to be expected because of continued degradation. Nonetheless, habitat supply for these fishes will be improved and created, with the expected gains modelled and monitored. Some fishes or life stages may benefit more than others from actions, and actual outcomes will be shown through continued monitoring, and model and equivalency improvements to better guide actions.

On the open coast, to the east and west of Toronto Harbour, the stone-hooking industry removed large quantities of boulder, cobble, and gravel from the lake bottom for use as building materials in Toronto (RCFTW Royal Commission of the Future of the Toronto Waterfront (1991b)). These removals destroyed spawning and rearing habitat for valued coldwater fishes such as Lake Trout, Lake Whitefish, Round Whitefish, and Cisco (Kelso et al. 1996; TRCA Toronto and Region Conservation Authority (2016)). Also, the network of smaller embayments and wetlands that linked upland areas to lake habitats were either disrupted or destroyed by channelization, infilling, and shoreline hardening, as urban areas were expanded along the waterfront and into the lake. However, even given all these stressors, the natural productivity of the nearshore area along the north, open coast of Toronto on Lake Ontario is, and was likely, limited to predominantly coldwater species. It is a large great lake in a temperate climate, with frequent, coldwater upwellings and intrusions in summer (Murphy et al. 2011; Hlevca et al. 2015). Therefore, the natural productivity expected from open coast areas will the benchmark target for that ecotype; and the restoration of lost shoals in the open coast from stone-hooking is one habitat target set for the nearshore.

### Geographic (Spatial) Boundaries

The geographic boundaries for an integrated planning or habitat bank service area may be based on several considerations, including ecological. The area should be sufficiently large to account for human development and habitat management activities at an ecologically meaningful scale, but not be so large that costs and benefits are inequitably shared spatially (e.g., the whole of Lake Ontario). Setting the area’s spatial extent should consider existing jurisdictional and governance boundaries, but focus on conservation boundaries, natural landscape features (e.g., watersheds and rivermouths), and other ecological factors affecting the fisheries productivity of the systems (forage base distributions, fish communities, and fisheries management units). For example, one ecological criterion to help define spatial extent could be the range within which an average, non-migratory, fish subpopulation may function, but its placement should be guided by natural largescale, eco-zonation (Yurista et al. 2012; Chu et al. 2014).

**Fig. 2:**
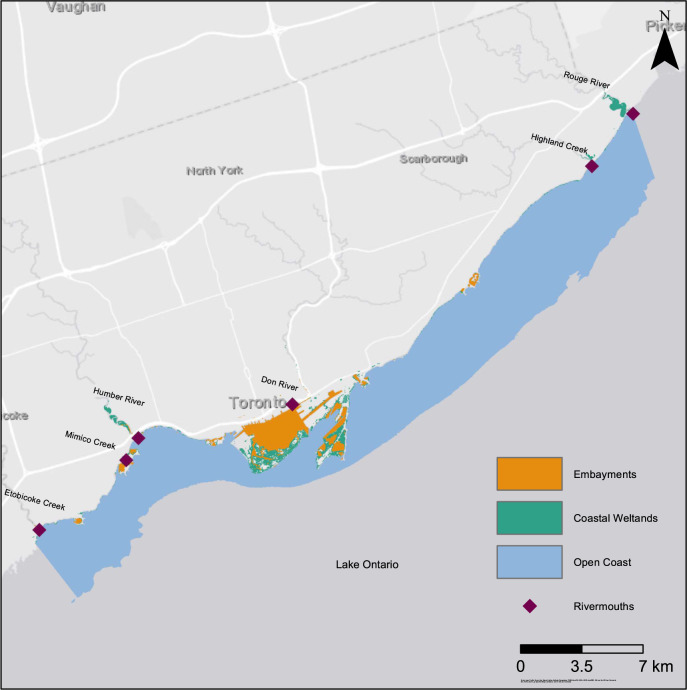
Map of the integrated planning area used for the Toronto region’s nearshore. Proposed boundaries are: the east and west land boundaries of the TRCA extended into the water to a southern boundary at the lake-floor elevation contour of 44 m ASL (roughly 30 m depth at reference lake level datum, (IGLD 1985), and extending upland to a northern boundary of 77 m ASL. The nearshore planning area include srivermouths (purple diamonds), open coast (light blue), embayments (orange), and wetlands (turquoise).

As such, the boundaries of the proposed Toronto region nearshore, integrated plan area in the northwest quadrant of Lake Ontario were based on existing jurisdictional, resource management, and ecological zones (Fig. 2):

1. The Toronto and Region Conservation Authority (TRCA) limits were chosen as practical and informal eastern and western boundaries for the integrated planning area; this area includes rivermouth and other nearshore ecotype habitat features, like wetlands;
2. The southern boundary of the proposed integrated planning area was defined as the lake-based, elevation contour of 44-m ASL, roughly the 30-m depth contour (International Great Lakes Datum 1985: CCGLBHHD 1992) and likely the maximum thermocline depth; and,
3. The northern, upland, or inland boundary was defined by the 77-m ASL elevation, which is slightly higher than the long-term record for lake water levels (i.e., roughly the 100-yr high water level of 76.3-m ASL). The upland boundary could be expanded if a larger riparian zone was considered an important natural feature to include, in an aquatic sense, beyond the long-term flood zone, which can vary depending on local slope (e.g., bank versus flood plain).

These boundaries make the habitat integrated planning area readily manageable within the broader landscape because they fall within existing jurisdictional watershed boundaries and are also within a similar hydrodynamic zone of Lake Ontario. Also, the TWAHRS applies within the TRCA limits, so the extent is also compatible with these local watershed and municipal boundaries, providing goal and target consistency.

The 44-m ASL elevation contour or 30-m depth corresponds to a liberal limnological definition of the nearshore or littoral zone, still accounting for variability in maximum thermocline depth (sensu Wetzel 2001; i.e., above the profundal zone). This elevation also corresponds to the ecological and physical features of the Toronto scarp, located roughly at the 30-m contour, beyond which lake depths increase rapidly offshore to 60- to 75-m depths within a distance of ~100 m.

At the upper limit of 77-m ASL, this boundary includes periodically flooded lake habitat, and some key transitional, upland riparian features. The high watermark of Lake Ontario is defined to be the upland extent of fish habitat; however the 80th percentile water level continues to be used as the upper regulatory extent of fish habitat in the Great Lakes, as a guideline (DFO Fisheries and Oceans Canada (2005)). This broad definition of the integrated planning area also includes some terrestrial areas that may be converted in future to regain aquatic habitats lost to extensive historic infilling. In downtown Toronto, this includes the land south of Front Street. For example, a proposed wetland reclamation, converted from land to aquatic on the Toronto Islands, would be considered a deposit to the aquatic accounts. Also, the upper elevation boundary includes some upstream areas of tributary mouths to the extent that they are influenced by lake level fluctuations. By including areas just above the 100-yr flood level (77-m ASL), rather than limiting boundaries to 75-m ASL (the 80th percentile of historic high-water levels), we capture the value of wetlands and intermittently flooded areas above the elevation previously used for regulatory purposes, but may be important periodic contributors to fish habitat supply or local natural processes.

In some instances, higher or lower elevation boundaries may be chosen for habitat banking purposes. For example, if riparian, stream, or wetland areas immediately above the high-water elevation, or features below the profundal zone, are shown to be essential for local fishery productivity, then these boundaries could be revised on a case-by-case project basis. However, once the total area and limits of the integrated habitat planning area have been agreed upon they should be considered permanent, or at least longer-term, otherwise ecological accounting may become complex and unwieldy with constant revisions and updates. This is good rationale for being generous in setting the boundaries and spatial extent at the outset.

### Ecotypes

We define ecotypes as distinct, major habitat types within the integrated planning area, to be used for mapping, accounting, and productivity calculation purposes. However, we recognize the whole system is interconnected and gradients exist between classes, therefore we advise liberal boundaries for productive ecotypes so they are not undervalued. Nonetheless, freshwater lake ecosystems generally have easily recognizable ecotypes (Minns and Wichert 2005; Chu et al. 2014). The key ecotypes assigned for area-based management of the Toronto waterfront include:

1. *wetland*: shallow areas dominated by emergent and submerged vegetation, often with constricted connections to adjacent open coast aquatic areas;
2. *embayment*: a bay or bay-like conformation defined by the Free Dictionary as “an indentation in a shoreline or coast forming a bay with intermittent or permanent connection to a larger body of water; bigger than a cove but smaller than a gulf.” (For our purposes embayments are relatively sheltered areas but do not necessarily have emergent vegetation like wetlands, though may have submerged vegetation if shallow;
3. *rivermouth*: the delta area or transition zone between the inflowing waters of a river and a receiving lake or river (Larson et al. 2013) where the local area is impacted by both riverine and lacustrine forces and gradients from the inflowing watershed;
4. *open coast*: defined here as the nearshore zone (<30-m depth) that is exposed to, and driven by, whole lake processes, and not sheltered, as with most wetlands and embayments; and,
5. *offshore*: open lake habitat where water depths are greater than the littoral or maximum epilimnetic zone depth (in this case >30-m depth). Offshore waters have also been defined here as those >5 km from the shoreline along open coasts (i.e., not within embayments) if the maximum depth of 30 m has not yet been achieved at this distance.

Ecotypes acknowledge that there are mesoscale combinations of abiotic and biotic features that often support differing biotic communities or assemblages and differing productivity levels (Walters et al. 1999; Wegscheider et al. 2020). The first four classes listed above are generally considered littoral or nearshore habitats. It is also generally accepted that maximum potential productivity per unit area is highest in wetlands and lowest offshore, with rivermouth, embayment, and open coast in intermediate rank order of productivity per area (Randall et al. 2004; Howell et al. 2012; Yurista et al. 2012; Chu et al. 2014). In the habitat accounting framework, the ecotypes must be assigned productivity scalar values to establish their equivalencies when trading habitat parcels among the various ecotypes. For example, wetlands typically support conditions preferred by warmwater and coolwater fishes at various life stages, while open coast, pelagic areas support coldwater fishes, but not all life stages, and which are less numerous per area. Wetlands are typically more productive per unit area than open coast habitats. However, their relative proportions may differ across the whole lake so overall total production may be similar because open coast habitat is common in most Laurentian Great Lakes.

Ecotypes avoid creating fine-grained classification systems that may require considerable resources to map accurately. Defining mesoscale classes in valuation systems and using productivity benchmarks are two techniques being developed in the Toronto area based on comprehensive sampling in at least three of the five ecotypes identified (Hoyle et al. 2018; TRCA 2018). A full regional-level spatial assessment is incomplete—however, the central waterfront is done (Leisti et al. 2020)— but barring a detailed evaluation of the status and relative productivity of the ecotypes locally. However, we have set up the preliminary account tables with some accurate, but other notional and expert inputs, for illustrative purposes (Tables 1–5).

**Table 1:** The potential productivity index (PPI) expresses the relative maximum ongoing productivity for ecosystem types

	Ecotype				
	Rivermouth	Wetland	Embayment	Open Coast (≤30 m)	Offshore (>30 m)
PPI	1.5	2.0	1.0	0.5	0.25

The values are speculative at present and loosely based on an HPI developed for fishes by Randall et al. 2004 and unpublished data from Hoyle et al. 2018. For the purposes of the Toronto region nearshore integrated planning area, terrestrial ecotypes are not considered as valuable to aquatic habitat in this prototype yet

**Table 2:** The percent weights proposed for HEAT assessments for weighted suitable area calculations (WSAs), thermal guilds and life stages are differentially weighted across ecotypes based on differences in their relative importance to those fish groups

Thermal guild	Life stage	Eco-class				
		Rivermouth	Wetland	Embayment	Open coast	Offshore
Cold-water	Spawning	6	4	7	22	20
	Nursery	7	4	6	20	22
	Juv + Ad	8	2	5	18	24
Cool-water	Spawning	11	11	15	8	8
	Nursery	11	10	17	12	12
	Juv + Ad	11	9	17	10	10
Warm-water	Spawning	15	19	11	2	1
	Nursery	16	22	11	4	1
	Juv + Ad	15	19	11	4	2

Percent weights are notional at present

*Juv* + *Ad* juvenile and adult life stages

**Table 3:** A notional baseline balance sheet for aquatic habitats and their relative productivity in the Toronto Nearshore integrated planning area

Variable	Units	Terrestrial habitat^a^		Aquatic habitat ecotypes					Total
		Mainland	Islands	Rivermouth	Wetland^b^	Embayment	Open coast^c^	Offshore^c^	
Baseline	ha	130	80	30	160	830	14000	440	15,670
	%	0.8	0.5	0.2	1.0	5.3	89.3	2.8	100
Current	ha	Updated on regular basis after baseline complete							
	%								
Objective^d^	ha	157	157	78	784	1567	12426	439	15,670
	%	1.0	1.0	0.5	5.0	10.0	79.3	2.8	100
Aquatic habitat quality (Mean Suitability)									
Within ecotype (ha)	High (0.75)			60	80	50	65	50	9881
	Med (0.50)			10	15	40	25	30	3991
	Low (0.25)			30	5	10	10	20	1588
	WSA	0^a^	0^a^	12.50	27.50	43.75	17.50	71.25	–
Among	PPI	0	0	1.5	2.0	1.0	0.5	0.25	–
Baseline	PWSA	0	0	18.75	55.00	43.75	8.75	35.625	161.875

Baseline, current, and objective proportions were estimated for terrestrial buffer and aquatic ecotypes. Then suitabilities and productivity scalars were used in the final accounting of habitat supplies to begin the balance sheet

^a^terrestrial habitat within buffer zones is valued at 0 aquatic suitability in this approach, but could be calibrated and evaluated for trading

^b^Wetlands may be in rivermouth or embayment locations, but typically are not found in open coast areas

^c^Open coast is defined as ≤30 m depth or <5 km from shore; offshore is deeper than 30 m

^d^Objectives are just notional and finalized yet for the Toronto nearshore region

**Table 4:** A simulated habitat banking trade based on pre- and post- construction and offsetting scenarios within the Toronto waterfront fish habitat service area (integrated planning area)

		Pre-Scenario						Post-Scenario						Net PWSA	Deposit Portion
Action/Project	Bank transaction	Ecotype	PPI	Area (ha)	Suit	WSA	PWSA	Ecotype	PPI	Area (ha)	Suit	WSA	PWSA		
Conservation	Deposit	Land	0.0	1.0	0.0	0.0	0.0	Wetland	2.0	1.00	0.75	0.75	1.5	1.50	
Development	Withdrawal	Embayment	1.0	1.0	0.5	0.5	0.5	Land	0.0	1.00	0.00	0.00	0.0	−0.50	
Bank	Trade							Wetland	2.0	0.33	0.75	0.25	0.5	0.00	0.33

No offset ratios were used

*PPI* potential productivity index, *WSA* weighted suitable area, *PWSA* productivity-weighted suitable area

**Table 5:** Integration of the habitat trade into the overall habitat supply balance sheet for the integrated aquatic planning area

Variable	Units	Terrestrial habitat		Aquatic or Fish habitat					Total
		Mainland	Islands	Rivermouth	Wetland	Embayment	Open coast nearshore	Offshore	
Baseline	PWSA	0	0	45	320	830	7000	110	8305
supply	Area (ha)	130	80	30	160	830	14,000	440	15,670
Deposit	PWSA		0.0		+2.0				
	Area (ha)		−1.0		+1.0				
Trade	PWSA		0		+1.0	*−*1.0			
	Area		+1.0		+0.5	*−*1.0			
Bank	PWSA				+1.0				
remainder	Area				+0.5				
Updated	PWSA	0	0	45	322	829	7000	110	8306
supply	Area	130	80	30	161	829	14,000	440	15,670

Embayments are protected areas with <5 km fetch. Open coast nearshore is defined as unprotected areas within 5 km of shore and ≤30 m depth and offshore is >5 km out or >30 m depth

*PWSA* productivity weighted suitable area

Relative indices, or scalars, that broadly reflect potential productivity differences among ecotypes provide a basis for converting to a common currency for habitat trading that is ecologically based. For example, an infilled loss of open-coast habitat might be traded against the gains achieved from the restoration or creation of wetland habitat within the integrated planning area. The differences in maximum potential productivity (i.e., constructed wetland > open coast) are taken into account when determining the scaled habitat area required to offset the loss of open coast habitat. Other considerations in the transaction should include the relative availability of ecotypes within the integrated planning area and the underlying trade-offs made between specific fish guilds that may prefer one ecotype over another, or select habitat variables across ecotypes. This habitat selectivity and availability at the larger landscape and fisheries objective level were considered in developing desired targets for the integrated planning area.

For the Toronto waterfront case study, we used an index we call the *potential productivity index* (*PPI*) to reflect differences in maximum potential productivity among different ecotypes. This relative index reflects expected ecosystem-level productivity that supports different fish communities or fish productivity over differing environmental conditions. Based on relative production estimates from the literature, the current PPI assignments are notional and will need to be refined once local assessment data are analyzed appropriately (Table 1). A thorough review of available local scientific evidence on ecosystem and fish productivity rates in the various ecotype habitats will be needed to establish scientifically-defensible PPI values. However, all parcels of habitat within a single ecotype are not expected to achieve the maximum potential productivity. Variations in habitat features and levels of degradation in individual parcels determine the proportion of the maximum attained for each area or project by using the valuation method outlined below. Within-ecotype variation and how it could be incorporated into a spatial assessment is discussed in the next section.

### Valuation of Habitat Parcels within an Ecotype Area

Habitat conditions can vary among parcels within any ecotype. For example, thermal conditions in Lake Ontario embayments can range from warmwater to coldwater, depending on their connection and exposure to the open lake where upwellings are prevalent (Murphy et al. 2012a). Equally, some areas may be strongly degraded with concomitant reductions in productivity (e.g., polluted or highly disturbed areas) and others are not. Thus, while the assigned PPI values represent a scalar (i.e. exchange rate) for maximum potential productivity within an ecotype, it is still necessary to evaluate, or at least account for, the varying environmental or habitat conditions among microhabitat parcels within each ecotype when trading within or between ecotypes.

Within-ecotype trading is likely since individual development activities are often small relative to the size of contiguous areas belonging to one ecotype and may only affect some habitat parcels within one ecotype (e.g., a small infill within an embayment or an open coast area). Particular features within an ecotype may have higher value than others (e.g., coldwater spawning shoals in open coast areas, if limiting). There is also variation within each ecotype due to differing spatial configurations, parcel sizes, depth ranges, substrate types, vegetation types, and thermal characteristics. This variation must be considered as part of the habitat valuation. The potential combinations of habitat features within any ecotype, coupled with any additional environmental stresses (e.g., turbidity or pollutants), means that maximum productivity can only be realized under ideal circumstances within any ecotype and therefore the PPI is scaled by local habitat parcel conditions using weighted suitable area (WSA).

Habitat valuations typically involve one of two approaches (Minns et al. 2001; Airamé et al. 2003; de Kerckhove et al. 2008). First, a quantitative or qualitative index or relationship that incorporates the habitat-association strengths of fishes, or their life stages, is used. Only species that are present at a site or within the locality are used in the calculations (e.g., in calculating a habitat suitability index [HSI]). Secondly, spatial or aggregate estimates of various biological, physical, or chemical features in the locality are determined. Most often, physical features are the variables assessed, but all features comprise fish habitat, including water quality. The assessment results are used directly to establish fish-specific indices. The second approach is limited to pre-development assessment, but complete evaluations require both pre- and post-development assessments to gauge net change. Often, the features chosen are those conditioning or constraining an ecological function of an area (e.g., substrate cover affecting spawning activity; vegetation type or amount affecting larval density or food availability).

Finally, the calculation of a relative habitat supply for a parcel involves combining two elements: the parcel area and its estimated index or suitability value. Only one element is considered if an area is evaluated solely on its suitability or solely on direct areal estimates of a broader ecotype. Multiple variables collectively contribute to overall habitat supply; based on their local influences, they determine a parcel’s overall quality. Across the service area and ecotypes therein, habitat parcels collectively contribute to system and local properties, like diversity, total biomass, or maximum potential productivity. That heterogeneity and relative contribution is implicit to the evaluation of proposed development and offset/bank trades (Ferraro 2013; Randall et al. 2014).

In the Great Lakes area of Ontario, DFO and project proponents have used various evaluation tools—e.g., HAAT (Minns et al. 2001), HEAT (DFO Fisheries and Oceans Canada (2019)), pHabSim (Milhous and Waddle 2012), or custom habitat evaluation procedure (HEP) or HSI approaches—for assessing habitat losses and gains resulting from development and restoration activities. HAAT (Habitat Alteration Assessment Tool) used vegetation, substrate, and depth associations of freshwater fishes and has been reprogrammed and upgraded as HEAT (Habitat Ecosystem Assessment Tool) to reflect relative guild differences in habitat preference and suitability that cannot be captured by only vegetation, substrate, and depth associations. HEAT allows for different fish guild or life stage weightings in suitability and supply calculations, if desired. This recognizes that each ecotype supports certain habitat guilds, life stages, or fish assemblages more than others and can be used to differentially weight development and restoration proposals based on fisheries objectives. In HEAT, regional or customized fish species lists—based on the project’s location and the relative weightings of habitats based on fisheries priorities (i.e., by guild, life stage, or other factor)—are used to value habitat subclasses within the project’s area of influence, based on regional fish-to-habitat associations.

In the HEAT scenarios for the Toronto waterfront, different fish guild weightings (exchange rates) were assigned for each ecotype (Table 2). Coldwater guilds were given higher weights in open coast and offshore areas, warmwater guilds were assigned higher weights in rivermouth, wetland, and embayment areas, and the coolwater guild was given intermediate weights in all ecotypes. Weightings can also be used to reflect local or regional fish community goals, like those within the TWAHRS, into transactions. For example, on the Toronto waterfront, increasing warmwater nursery habitat is a core goal given that so much of that ecotype and its associated habitats were lost as Toronto expanded into the lake via infilling of the historic Harbour and surrounding wetlands.

Within each ecotype, computations are performed on the habitat parcels to estimate WSA (WSA = Σ parcel areas × parcel suitabilities) and then modified using the ecotype-specific guild weightings. At this point the measure can be considered a suitable-habitat equivalency that is weighted by fisheries goals or guild preferences. The resulting WSA values are then multiplied by their ecotype PPI values to calculate the *productivity-weighted suitable area* (*PWSA* = PPI × WSA) to obtain a relative productivity equivalent across the whole region (for more detailed equations, see Abdel-Fattah et al. 2021). For example, warmwater fish guilds have higher weights in the wetland ecotype while coldwater guilds are valued more in the offshore or open coast ecotype, thereby recognizing differences in fish thermal selectivity of the ecotypes. In future, HEAT will explicitly incorporate temperatures into habitat calculations such that guild weightings may no longer be needed to account for thermal differences across ecotypes. However, guild weightings for assigning higher priority to specific guild or life-stage habitats can prioritize for regional or integrated plan area resource management priorities.

### Natural Capital Reserves

Once a full survey and accounting of the habitat supply or natural capital (e.g., Gertzen et al. 2012, Hooper et al. 2019) within a habitat banking service area is completed, some areas can be set aside a priori as protected natural capital reserves, and no longer be available for trading. Only the areas remaining outside the reserves would be accessible for trading purposes. The reserve areas should consist of high quality parcels (both natural and restored) across the ranges of habitat types and ecotypes present in the integrated planning area. With proper planning, the accounting for natural capital reserves can support large-scale management goals by transferring net gains to reserves, possibly recovering historical losses.

In relatively degraded regions like the Toronto nearshore, any remaining natural or restored areas should be automatically set aside as reserves. Further additions to the reserves from net gains achieved via restoration and offsetting or banking activities would become a primary policy aim for the integrated plan to sustain momentum towards maximizing and sustaining the potential productivity of that area (Koops et al. 2013). Habitat banking used in an area-based management approach such as this allows orderly planning of reserves over time, rather than achieving those goals via piecemeal site-by-site actions. Having pre-existing CPR plans will facilitate habitat bank arrangements if service areas are equivalent to integrated planning areas.

In Toronto, the TWAHRS is a semi-quantitative CPR plan and requires further quantitative work to establish the full ecological accounting system envisioned here. A fully realized CPR plan would depend on identifying habitat parcels that represent critical and/or limiting habitats, and recognize that less abundant ecotypes such as wetlands and rivermouths might not be ideal as habitat banks used for offsetting purposes if a net gain is desired. These reserves would instead be protected for their contributions to the future fisheries or aquatic productivity and biodiversity of the area. For example, the remaining emergent wetlands and submerged aquatic vegetation areas could be identified in the integrated plan as reserves from the outset, and be continually added to until an overall target for vegetated area is reached.

### Habitat Transactions

As with financial banks, the main habitat transactions are deposits, withdrawals, and transfers. In an area where ecological restoration or banking is the focus, deposits are required first to achieve a positive balance through habitat creation or improvement. Adverse impacts to habitat are withdrawals from the plan area and traded for some portion of the available accrued deposits to counterbalance a development project’s residual impacts. Time lags for the newly created habitat to become ecologically functional may become part of the accounting. Waiting for this functionality to be achieved before allowing withdrawals from a particular deposit is akin to placing a hold on a financial deposit until the cheque clears. In rare circumstances, transfers of available deposits may offset residual impacts from a development project outside of a habitat bank’s integrated plan area.

#### Deposits

Planning habitat deposits involves several steps in the proposed scheme:

1. Ideally, a series of deposits should be identified and fully implemented before withdrawals begin so that the habitat banks or restoration projects have a positive balance from the outset and show net gain towards performance targets, before further losses are incurred. But this rarely happens in reality in a large city.
2. Habitat parcels (outside of reserves) that (a) have restoration or enhancement potential (i.e., can improve in habitat quality), or (b) might be used to create habitat (e.g., by converting terrestrial into aquatic habitat, if appropriate) and realize a gain in quantity are identified.
3. Habitat parcel values are assessed using their relative quality within an ecotype (i.e., by using HEAT or other approach in units of WSA = Area × Suitability). Habitat supply by parcel is then scaled by the PPI of each ecotype to bring all habitat parcels to an equivalent base unit across ecotypes (i.e., productivity-weighted suitable area or PWSA = WSA × PPI). For terrestrial parcels identified for potential aquatic habitat creation projects, their initial PWSA is assumed to be zero in a fish habitat sense. However, a more comprehensive planning approach might include valuation of aquatic and terrestrial ecotype areas to address those trade-offs more explicitly.
4. The improvement or creation of the habitat parcels is planned and financial costs are estimated along with PPI. Measurable assessment benchmarks will be needed to ensure that both functional habitat and productivity targets have been achieved. This necessitates some level of short- and long-term monitoring and evidence-based criteria for use in planning and costing.
5. Once the deposit construction is complete and the ecological performance targets have been met — time lags from habitat creation or enhancement to ecological functionality are accounted for here—the net PWSA can be deposited into the integrated planning area’s balance sheet, and become available for trade with a future offset withdrawal, if needed.
6. The full financial costs of the habitat deposit, including costs for planning, construction, and long-term monitoring and assessment (needed to demonstrate the target productivity is achieved and sustained) are accounted for in a financial counterpart to the ecological habitat supply accounts. The financial methods are not presented here, but we feel it is important to mention. Those costs should also reflect anticipated inflation and other economic factors associated with construction, assessment, and monitoring, and possibly other valuations of the habitats (e.g., recreational use, carbon, biodiversity). However, only one value is traded even though the deposit may have multiple benefits. This avoids stacking, or ecological double dipping (Gillenwater 2012)

#### Withdrawals

There are several steps to complete a withdrawal from the integrated planning area’s habitat supply:

1. When a proposed development project is projected to impact aquatic habitat, efforts are first made to avoid the potential adverse effects using the mitigation hierarchy. Where possible, remaining effects are minimized through on-site mitigation measures: before, during, and after construction. Unavoidable adverse effects become candidates for offsetting deliberations.
2. After avoidance, mitigation, and offsetting have been decided upon, habitat supply assessments (via area, WSA, PPI, and PWSA assessments of habitat parcels) are undertaken to evaluate net change at different levels of a project using projected scenarios. The aim is to maintain a positive balance in the service area. If the evaluation indicates losses cannot be offset onsite, or located elsewhere in the integrated planning area in a timely fashion, additional withdrawals from the habitat bank could balance residual effects but would ultimately reduce gains.
3. Once agreed upon, offset units and their habitat types and ecotypes are used in calculations of pre- and post-development comparisons to show equivalency balances. Calculations include any other ratios that need to be applied (e.g., time lags, discussed at step 5 below). One or more habitat parcels or ecotype areas within the habitat bank (near or removed from the development construction site) may be considered as offsets for development withdrawals—depending on CPR plans, such as the TWAHRS—and brings context to actions based on local ecological needs and knowledge over the landscape scale (Prime et al. 2013).
4. The final amount of offset PWSA units for trading against adverse development impacts are withdrawn from accrued deposits (effectively creating a banked project assigned to the development project). The traded deposits are ‘moved’ into the integrated planning area reserves and are no longer available for offsetting. Additional habitat projects not yet constructed can be designed and then deposited for later withdrawal depending on the number and magnitude of future development projects anticipated and the negotiated timing of deposits to collective habitat banks. This landscape-based approach may require third-party banking be allowed, but with proper planning has been effective under current, single-party regulatory requirements in Canada.
5. The timing of impacts (e.g., construction or timing windows; DFO 2018) and lag times between deposits and withdrawals should be considered in the ecological valuation to balance actual accrued losses and gains over time (Minns 2006). The pro-rating of deposits, (i.e. the opposite of discounting impacts from projects, based on time lags, before offsets are built and functioning) could be critically evaluated as an important aspect of project and offset timing, and as an incentive for habitat banking. CPR plans and financial accounting within the habitat service area should also be considered in timing aspects, as some habitats are more difficult to construct (e.g. wetlands) and costs continually inflate.
6. The proponent pays the economic costs to design, construct, monitor, and maintain the habitat deposit until maturity, or in perpetuity, once decisions on offsetting are finalized with regulators. If third party banking is allowed, then costs will be transferred between banker and proponent. In Canada, currently this in not allowed so informed planning by proponents is key to avoiding random acts of offsetting.
7. Monitoring data from all projects are used to ensure that biological and physical habitat endpoints are behaving and improving as anticipated. Multiple valuation methods, such as biodiversity in combination with productivity, can inform preferred alternatives as well. Adaptive management of habitat changes (both developments and offsets) can be used to modify the CPR plan and update valuations accordingly as more experience and knowledge of different types of habitat loss and modifications are gained over time from the analysis of pre- and post-monitoring data.

#### Transfers

We have already discussed intra-service area trading—deposits and withdrawals within the same integrated planning area, a foundation of habitat banking—but there may be situations where an inter-service area transfer could be considered. Such a transfer may be needed when there is an unavoidable, anticipated loss with no, or not enough, restoration or creation potential locally. In standard approaches, efforts are made to achieve no net loss locally, but some areas may lack CPR plans, may not have a habitat bank option, or may lack the opportunities to enhance native biodiversity or fisheries production (Minns 1997). In these instances, IP groups might consider larger-scale but connected, management areas and their objectives for trades (i.e., transfers in this case) that cross the original, defined service area or integrated planning area boundaries.

For the Toronto nearshore service area, a larger scale might include lake-wide fish community objectives (Great Lakes Fishery Commission, Stewart et al. 2017), lake-wide nearshore management plans (ECCC and USEPA 2018), or international habitat strategies developed for the whole of Lake Ontario (LOBSWG Lake Ontario Biodiversity Strategy Working Group (2009)). The out-of-area deposit could still benefit the local area by creating or enhancing broader fisheries production in a different but connected coastal or fisheries management unit. Many fish use may use a broader scale at for migrations or excursions. Priority restoration needs for particular fisheries species can sometimes be achieved by connecting high quality areas and functions, or restoring known degraded areas. The possibility of inter-service area transfers recognizes that some ecological functions operate over larger spatial scales, and fish community goals may not be optimally achieved within a single, local integrated planning area (i.e. just local CPR plans). However, overuse of the inter-bank transfer option could result in further degradation within some areas if offset benefits are accrued elsewhere; this is a definite consideration in the initial designation of the integrated planning/service area boundaries. Hence, a precautionary approach for the use of inter-bank transfers is advised, and full, advance consideration of the appropriate management scale is so important.

#### Financial and governance considerations

In this eco-accounting framework, the planning, construction, and monitoring of offset deposits falls to the banker or proponent as per regulation. In our case study, AHT is the integrated planner coordinating trades and advising within a set service area. In addition to ecological costing, the financial costing of the trade for the developer includes the full costs for the portion of the created or enhanced project used in trading (i.e., costs of planning, construction, monitoring, inflation, fees, etc.). This amount is paid directly for creating future habitat bank deposits or to support required for ongoing monitoring and assessment activities. Basic monitoring and assessment at the development site will still be the responsibility of the proponent. In the Toronto example, AHT is well established with expertise in habitat creation, restoration, and monitoring within the integrated planning area and has ongoing science support to guide decision-making (Prime et al. 2013). Clarification on the role of a third-party banker is needed with respect to the ecological and financial trading activities required for the area. A governance model would need to be developed to ensure that different banking activities were handled by quasi-independent agents or groups. We recommend that a separate financial and governance outline consistent with existing policies be drafted to complement this ecoaccounting framework.

## Results

### Simulated Application to the Toronto Region Nearshore Integrated Planning Area

We introduced the issues faced by the Toronto region nearshore and provided context for the need for integrated planning and accountability. To initiate the service area for trading, subareas in the bounded region were assigned to one of the ecotypes, then within each ecotype, the quality of all habitat parcels was evaluated. High resolution spatial data were not available for most of the open coast in the Toronto Region, so a crude baseline has sufficed for illustrative purposes. Ideally a complete habitat supply analysis of the service/IP area would be conducted (Bakelaar et al. 2004; Remillard et al. 2021), but this can be developed gradually so that trading losses for deposits can begin with a rudimentary assessment.

Here, we estimated areas by ecotype and assigned high, medium, and low habitat suitability categories with approximate mean suitability values of 0.75, 0.50, and 0.25, respectively, based on regional fish-to-habitat association information in HEAT (Table 3). Within each ecotype, a crude weighted suitable area (WSA = Area × Suitability) was calculated. Then the summed WSA values were scaled by the PPI weights (Table 1) for each ecotype to produce standardized productivity-weighted units, or PWSA values. For now, terrestrial buffer areas (i.e., those above the high-water datum) were assumed to have zero aquatic suitability. Both raw areas and PWSAs can be summed to produce a statement of relative habitat supply reflective of the overall maximum potential productivity in the integrated planning or service area. Goals are approximated, and based on TWAHRS objectives; coastal wetland area target is best known.

As noted, the first transaction in an established service area is the bank deposit (Table 4), which should occur prior to the first withdrawal and ideally be constructed sufficiently in advance to have achieved the expected potential habitat supply, biodiversity or productivity of the ecotype it represents. The weighted size of the deposit should be larger than any anticipated individual withdrawals in the near future to account for offset ratios, if required for time lags and uncertainty, in future trade calculations (Minns and Moore 2003). Here, AHT chose a 1-ha parcel of land adjacent to existing high-quality, aquatic habitat for conversion into a wetland. This deposit resulted in a net PWSA gain of 1.50 units (Row 1, Table 4). In our example, a hypothetical development activity elsewhere on the waterfront involved an unavoidable loss of an embayment parcel to infilling. The 1-ha embayment area had a moderate suitability and resulted in a net PWSA loss of 0.5 units (Row 2, Table 4). To offset the embayment loss, a portion of the wetland deposit (0.33 PWSA units or 33.3% of the area) was traded against the infill loss, resulting in a net-zero PWSA traded; note, no offset ratios were applied (Row 3, Table 4). The balance of 1.00 PWSA unit remaining from the original wetland deposit remains and may be used as a banked offset in another trade for an impact in the future, or used as a net restoration gain in this degraded area. However, current regulations would only permit one proponent to build and use the banked deposit from the project.

Given the baseline habitat supply statement for the habitat bank (Table 3) and the sample trade transactions (Table 4), a habitat supply statement can now be updated (Table 5). The total supply of wetland habitat shows an overall PWSA increase of 1.50 units (i.e. an area of 1-ha with 0.75 high suitability gained with an PPI of 2). The terrestrial portion within the service area loses no PWSA units (i.e., it is currently assessed at 0 aquatic value) but does lose 1 ha of actual area in the tracking tables. The unused deposits are tracked as a subtotal in the overall supply showing what offsets may be available for future development needs of the proponent (or new deposits that may be required to maintain an adequate positive balance).

When a detailed inventory of habitats is completed for the IP area, better areal estimates of each ecotype and their relative valuations will be updated and the habitat reserves identified explicitly. This will also include the net benefits of the offset project being assigned to the reserves for long-term conservation and protection (i.e., as part of a long-term CPR plan). Currently, the accounting framework as outlined has only partially been implemented.

## Discussion

The ability to expand the scope of habitat supply accounting from the project/site level to an integrated plan landscape level using an ecological framework for habitat banking has been demonstrated here. The Toronto region waterfront is a relatively well-studied shoreline with a substantial recent history of efforts to restore and create aquatic habitats to offset the long-term accumulation of losses incurred over the last two centuries, and in anticipation of development needs for the 25-year plan for revitalization of the waterfront by the City of Toronto and Waterfront Toronto. Past and ongoing efforts to assess fish communities and their habitats have produced a wealth of understanding and experience in Toronto (Prime et al. 2013), particularly with respect to restoration actions (Cooke et al. 2016; Brooks et al. 2017; TRCA Toronto Region Conservation Authority (2018)).

Full implementation of the systematic and quantifiable approach outlined here will likely need some additional detailed inventory of habitats, though this information can be gathered gradually, along with accumulating habitat deposits and trading for unavoidable adverse effects. Currently, despite being well studied, this information is not available for the full extent of the Toronto region service/IP area. Ideally, a concerted effort to map the basic habitat features of the area in a reasonable time frame would produce a better baseline estimate. New remote-sensing imagery (e.g., lidar) and the analysis of its reflectance (e.g., vegetation or substrate type) may help. Finally, the development community and public support for restoration and enhancements are already embodied in the TWAHRS document (TRCA 2003), laying out goals and objectives with active participation and endorsement from large proponents like the City of Toronto and Waterfront Toronto. Together these factors provide a strong basis for implementing a Toronto region nearshore integrated planning area with habitat banking to achieve fish biodiversity and fisheries production goals. And which may be able to, in the short-term, to expand into wildlife and other ecosystem services valuations in nearby terrestrial areas because of ongoing complementary work.

In the proposed ecological accounting framework, we adapted existing tools and incorporated general scientific knowledge about productivity differences among ecotypes, allowing a common habitat equivalency/currency, beyond weighted suitable area, to be modified. This common currency can be applied to habitat parcels across the complete range of aquatic ecotypes present in the Toronto regional nearshore. The methodology, framework, and habitat valuations can be updated as the knowledge base and tools are enhanced over time; this should be an adaptive iterative process, both locally and more generally. Habitat supply accounts can be updated on a regular basis, perhaps every 3 to 5 years as part of routine reviews of the habitat bank’s operations. Also, the natural variability in the baseline habitat features should be captured so that the ecological value of transitional and ecotone habitats between ecotypes are also captured in the valuation.

Ideally, the nominal estimates for the PPIs shown here will be replaced with actual fish production or primary productivity units, but still account for differing community composition and ecosystem dynamics across ecotypes (Randall and Minns 2000; Randall et al. 2017). The role of natural variation, especially temporal, in fisheries productivity within an ecotype (notwithstanding habitat differences already accounted for) still needs to be considered in this offsetting scheme. Bouvier et al. (2009) explained variance in wetland fish indicators from habitat factors at different scales. The natural variance across wetlands related to habitat quality (local scale) and habitat connectivity (broader scale) can help bracket expected fish production in the wetland ecotype. Similar analyses for the other ecotypes can be undertaken to improve PPI estimation.

Standardized monitoring will be an essential activity to ensure that CPR goals are met and to locally calibrate PPI values. The more monitoring data is shared, the less long-term monitoring of all variables is needed as ecotype knowledge becomes fine-tuned. Monitoring can demonstrate to habitat bank administrators the continued value of the deposited and reserved habitats, although this site-specific information needs reconciliation with the broader or more natural landscapes. Progress toward the fisheries and biodiversity goals for the IP service area can be tracked through effectiveness and regional monitoring (Granados et al. 2012; Lapointe et al. 2013; Hoyle and Yuille 2016), with the latter coordinated across agencies invested in landscape-scale (e.g., TRCA) or area-based management (e.g., Ontario Ministry of Natural Resources and Forestry, Environment and Climate Change Canada) within the integrated planning area.

Both monitoring and accounting will provide detailed, transparent documentation of IP area activities and progress towards regional goals; this is a sound management approach regardless of whether an official service area is declared. An evolving CPR plan for the Toronto area can be expected because the TWAHRS is a living document intended to be continually updated. Updates to plans and continued movement of areas to accounting reserves will affect future transaction opportunities between ecotypes in the integrated planning area. For example, to achieve overall habitat goals, wetlands or embayment habitat may be increased by converting open coast, but avoiding as much loss (e.g., infills) as possible. Ironically, to achieve these goals, it is necessary to include a loss in the accounting from infilling to recreate the lost embayments and wetlands in an area where reclaiming lake from historical land infilling (or from natural shorelines) is near impossible. Overall, the goal is for a targeted positive balance in habitat supply, reaching ecotype goals, and also reserves of natural capital to not duplicate historical cumulative losses.

To become fully operational, the financial accounting (how dollars are actually traded) and governance system (how decisions are made) for any integrated spatial aquatic planning will have to be fully developed in parallel with this proposed ecological accounting system. TRCA and other similar groups already have a good appreciation of the immediate costs of creating, enhancing, and restoring habitats based on their involvement in the intensive and extensive activities along the Toronto waterfront. It will be important to make sure the full financial costs—including long-term monitoring and maintenance costs for the accumulated deposits and any longer-term science support required for adaptive science and management—are estimated accurately for different habitat modification and creation activities. These costs, including estimates for different offsetting options, could be outlined in a separate financial framework. Governance models that separate or lump the different activities within the habitat banking system and their regulatory needs will also be required, and there are good examples (Bovarnick et al. 2010; Bull et al. 2016) and some guidance from policy (DFO Fisheries and Oceans Canada (2013), DFO Fisheries and Oceans Canada (2019)).

Regarding governance, AHT— with both science and regulatory groups sitting at the table—is well positioned to provide timely and effective aquatic habitat guidance within the Toronto IP area. Similar entities, and regional and local groups have been formed to address landscape-scale habitat and resource management needs, and many examples from U.S., E.U., Africa, Oceania, and Asia exist, which could serve as a model for similar approaches in Canada at appropriate regional scales (de Kerckhove et al. 2017). Further work will also be required to address the social and legal implications of the service area, as well as the relative contributions and responsibilities of the multiple agencies, developers, and stakeholders involved. As designated deposits are to be used to offset historical or new residual impacts on fish and fish habitat, there needs to be agreed upon standards for their stable state and condition to ensure long-term sustainability and resiliency, but given local expectations of variability (e.g., Murphy et al. 2011, 2012a, b). At present, integrated planning focusses on lacustrine habitats along the Toronto waterfront, with specific goals laid out in the TWAHRS. In our example, existing wetlands are protected and would therefore be set aside as reserves in the accounting scheme. An additional target for wetlands would be to recover as much of this ecotype lost historically, by converting other ecotypes (including terrestrial) to create wetlands, as outlined under the reserve section of this CPR plan. The formal quantification of those goals in the accounting framework ensures that trading reflects those goals more directly, and also requires considering their compatibility and sustainability in the overall integrated planning service area.

In the future, the habitat banking service area/integrated planning area could be expanded to include all tributaries and watershed habitats—including inland/terrestrial ecotypes (i.e., riverine, upstream wetlands, and their riparian zones)—to fully appraise and value riparian terrestrial trades with aquatic ones. Under the current scheme those trades are undervalued, especially for semi-aquatic species (e.g., herptiles). There is also scope for the accounting scheme to be expanded beyond fish productivity units to consider habitat biodiversity (Morrison et al. 2001) and habitat and ecotype connectivity (Bouvier et al. 2009) in the valuations. Metrics would need to be combined, and chosen to avoid benefit stacking (if taking a bundled approach), using multiple metrics to evaluate collective benefits (Cooley and Olander 2011). Using a common currency, or bundling of currencies, for trading, whether using single or multiple metrics, would require an augmented scheme be developed. Perhaps, expanding upon currently unitless determinations like weighted suitable or usable areas (Minns 1997; Minns et al. 2001) to those used in Ecologically and Biologically Significant Areas (DFO 2004, Randall et al. 2014) or Marxan-type approaches (Airamé et al. 2003).

For now, AHT plans to use the ecological accounting system outlined here for the Toronto waterfront, but iteratively make improvements as the methods and habitat equivalents are refined. This approach has potential for broader scale application across the Great Lakes for habitat management where landscape-scale service areas are appropriate for CPR plans to benefit fish populations, communities, and ecosystems, while allowing development to continue and implementing broader restoration goals beyond site-by-site and project-by-project considerations.

## Supplementary Information

Supplementary Information
