# Supplementary material for: An Ecological Accounting System for Integrated Aquatic Planning and Habitat Banking with Case Study on the Toronto Waterfront, Ontario, Canada
Source: Environ Manage. 2022 Feb 2;69(5):952–71. doi: 10.1007/s00267-021-01531-5 (PMC9038875; doi:10.1007/s00267-021-01531-5)
Supplement: Supplementary file 1 — Supplementary Information [file 267_2021_1531_MOESM1_ESM.pdf]

Supplement 1: Glossary of terms used in the manuscript

Title: An Ecological Accounting System for Integrated Aquatic Planning and Habitat Banking with Case Study on the Toronto Waterfront, Ontario, Canada

Authors: Susan E. Doka* [1], Charles K. Minns [1,2] , Brent G. Valere [3], Steven J. Cooke[4], Rick J. Portiss[5], Thomas F. Sciscione[5], and C. Alwyn Rose [6]

Journal: Environmental Management

***corresponding author**: susan.doka@dfo-mpo.gc.ca

Corresponding author affiliation:

[1]Fisheries and Oceans Canada, Great Lakes Laboratory for Fisheries and Aquatic Sciences, 867 Lakeshore Rd, P.O. Box 5050, Burlington, Ontario Canada L7S 1A1

Supplement 1: Glossary of terms used in the manuscript

AHT: Aquatic Habitat Toronto; A consensus based partnership between agencies with a vested interest in the improvement of aquatic habitat on the Toronto Waterfront through the implementation of the Toronto Waterfront Aquatic Habitat Restoration Strategy (TWAHRS) (TRCA 2003, Prime et al. 2013).

Biodiversity offset: measurable conservation outcomes resulting from actions designed to compensate for significant residual adverse biodiversity effects arising from project development after appropriate avoidance and mitigation measures have been taken. The goal of biodiversity offsets is to achieve no net loss and preferably a net gain of biodiversity on the ground with respect to species composition, habitat structure and ecosystem function and social and cultural values associated with biodiversity (BBOP 2012).

Bundling: receiving a one-time credit for multiple ecosystem services for one offset project (e.g. carbon offset, habitat offset, biodiversity offset etc. are bundled); banking is effective if each impact is offset only once and not traded separately which is considered ‘double dipping’ or stacking (Cooley and Olander 2011).

Natural capital reserves: defined here, as a collection of ecologically or biologically significant areas (EBSAs) that are identified during conservation, protection and restoration planning (herein, CPR plans); these reserve areas cannot be traded; reserves would also include any conservation/restoration or offset project area that has reached its full potential (which may not be equivalent to natural).

Conservation project: a work, undertaking, or activity that is carried on by a proponent for the purpose of creating, restoring, or enhancing fish habitat within a service area in order to acquire habitat credits (*Fisheries Act*); additionally, results in a net gain of fish habitat or fish productivity could be a restoration action recouping for historical losses or could be used to offset an impact through banking a deposit against future harm from a development project.

CPR (conservation, protection, restoration) plans: as defined in this paper, a landscape scale strategy for conservation, protection, and restoration of aquatic habitats and species where important areas are identified and protected, conservation practices are used to achieve no net loss, and restoration actions for impacted species and habitats are identified to achieve net gain.

Currency: in the context of ecological and habitat accounting frameworks, the ecosystem currency is based on a standard ecological measure (e.g., fisheries production or productivity in kg/ha/yr or habitat supply in hectares or weighted suitable area in suitable area-equivalents, typically considered unitless), also known as an equivalent.

Deposit: a habitat gain measured by units of area, suitable area, or some equivalency that represents the functions that the habitat provides (e.g., productivity, fish density, biodiversity) entered into the integrated planning are ledger; a conservation project's value as habitat; also known as a habitat bank project or offset, sometimes conservation or restoration project or action.

Development project: a work, undertaking or activity that may result in a net loss of fish habitat or death of fish requiring an offset to recoup its impacts through either banking a deposit, or accounting for continued temporal losses by offsetting / depositing at a later date.

Ecotype: for our purposes, a higher level, generic hydrological classification within which habitat variables can be defined more specifically (i.e., in lentic systems we define rivermouths, embayments, wetlands, open-coast littoral areas, and offshore profundal areas as different ecotypes).

Ecological accounting: a system where ecological or natural capital debits and credits are tracked using standardized metrics and valuation (e.g., WAVES = wealth accounting and the valuation of

ecosystem services; SEEA = system of environmental – economic accounting; blue accounting = metrics to evaluate effectiveness of investments and programs in environmental management).

Ecologically or Biologically Significant Areas (EBSA): a designation tool for calling attention to an area that has particularly high ecological or biological significance, to facilitate provision of a greater-than-usual degree of risk aversion in management of activities in such areas (DFO 2004).

Effectiveness monitoring: sampling a suite of indicators after a conservation, offset, or restoration project to gauge its effectiveness; a goal is to ensure it is functioning at normal levels of production, or another indicator, typical for the ecotype or habitat that the offset replaced, or the natural features it was meant to emulate (i.e., the locale can reach its maximum potential productivity when possible); required under certain monitoring and reporting conditions of offset permitting (Smokorowski et al. 2015).

Equivalency: a common currency or measurement metric that is used to standardize for ecological accounting purposes (e.g., weighted suitable area [WSA]); especially for trading between habitat types that are unlike, when adverse effects in one are offset by another.

Fish habitat: water frequented by fish and any other areas on which fish depend directly or indirectly to carry out their life processes, including spawning grounds and nursery, rearing, food supply and migration areas (*Fisheries Act*).

Fish habitat bank: can be used for an exchange between natural capital destruction (withdrawal) and natural capital generation (deposit), with the generation being performed first. Frequently, deposits and withdrawals take place within a designated service area (Hunt et al. 2011). The deposits are areas where fish habitat has been created, restored, or enhanced through one or more conservation projects within a service area where the Minister has certified a habitat credit under paragraph 42.02(1)(b) of the *Fisheries Act*.

Integrated environmental or resource planning: an environmental policy instrument (a framework/process) to regulate land/marine use and productive activities, to protect the environment, promote the conservation and sustainable use of natural resources, considering land/[aquatic] use potential and land/[aquatic] degradation trends. It is considered by some to be the most appropriate policy instrument to harmonize human activities and environmental sustainability in the short, medium, and long term (modified from Lausch 2019).

Habitat account balance: the accumulated deposits and withdrawals within a service area, within a specific account, or the net gain or loss across a suite of projects or accounts within an integrated planning area (i.e., total deposits - total withdrawals).

Habitat credit: a unit of measure that is agreed to between any proponent and the Minister under section 42.02 that quantifies the benefits of a conservation project (*Fisheries Act*).

Habitat suitability index (HSI): an HSM (habitat suitability modelling or matrix) or HEP (habitat evaluation procedure) technique using simple mathematical expressions for calculating a unitless index of habitat quality as a function of one or more environmental variables; values can be mapped and analyzed to show areas of potential animal distribution. High quality habitat may provide high carrying capacity and support higher rates of growth, survival, or reproduction for a given species, whereas low quality or unsuitable habitat may have little or no carrying capacity. HSI methods are adapted largely from the U.S. Fish and Wildlife Service Habitat Evaluation Procedures (HEP) program but are widely adapted using more quantitative methods (de Kerckhove et al 2008).

Habitat suitability modelling or matrix (HSM): a statistically based tool for predicting the suitability of a habitat for a given species or assemblage based on known affinities, tolerances, or effects associated with environmental parameters and species distributions. (See also "habitat suitability index [HSI]")

HEAT—Habitat Ecosystem Assessment Tool: a tool used to standardize habitat equivalents using a habitat association method (original version = HAAT; Habitat Alteration Assessment Tool) for fish communities (see https://habitatassessment.ca/; Minns et al. 2001).

No net loss: the point at which the project-related adverse effects on biodiversity or fisheries are balanced by measures taken to avoid and minimize the project's adverse effects, to understand on-site restoration effectiveness, and finally to offset significant residual adverse effects, if any, on an appropriate geographic scale (e.g. local, landscape-level, national, regional) (UNEP - WCMC 2014; Bull et al. 2016, zu Ermgassen et al. 2019).

Mitigation hierarchy (avoidance, mitigation, offset, restoration): the order of actions to address projected impacts from development projects; efforts are made to avoid adverse effects first. When avoidance is not possible, then efforts focusing on mitigating effects, then offsetting, and finally restoration to original condition, as possible (Arlidge et al. 2018, DFO 2019a,b).

Offsetting: measures to counterbalance adverse effects on fish and fish habitat by maintaining or improving fisheries productivity after all feasible measures to avoid and mitigate adverse effects have been undertaken (IUCN 2016, DFO 2019); see also biodiversity offset.

Performance targets: a criteria used to establish whether a project or a program is functioning as intended; SMART targets are preferred (SMART = specific, measurable, achievable, results-based, time-bound; Doran 1981).

Project: defined here, based on DFO's *Fish and Fish Habitat Protection Policy Statement* (2019), as one or more existing or proposed works, undertakings or activities.

Residual impact: the remainder of a development project's adverse effects after avoidance and mitigation strategies have been implemented; the amount of impact that should be offset (see also "withdrawal" [serious harm]).

Restoration: broadly defined as the act, process, or result of returning a degraded or former habitat to a healthy, self-sustaining condition that resembles as closely as possible its pre-disturbed state. It is very common for this term to include improving or increasing the function or area of a habitat or an ecosystem. The term is broadly defined here to include creation, enhancement, and rehabilitation actions.

Service area: the geographical area that encompasses a fish habitat bank of one or more conservation projects, and within which area a proponent carries on a work, undertaking or activity (*Fisheries Act*).

Stacking: receiving multiple, separate credits for several distinct ecosystem services for the same offset project; not effective if the same parcel is reused to trade in multiple markets separately (not simultaneously/bundled), or one market multiple times (aka double dipping) (Gillenwater 2012).

Transfer: a deposit (i.e., a conservation project or offset) made in one service area is used to counterbalance or offset a withdrawal (i.e., the residual impact from a development project) in a different service area. Policies should be checked on transfers; they may only be considered if no deposits can be found within a service area, and may need additional justification.

TWAHRS – Toronto Waterfront Aquatic Habitat Restoration Strategy: a collaborative strategy to maximize the potential ecological integrity of the Toronto waterfront through restoration of lost features (TRCA 2003).

Weighted suitable area (WSA): an indexed area representing the capacity of a habitat patch to support the species and life stages being considered, expressed as actual area or proportion of habitat area predicted to be usable; an equivalent area of high suitability; a production scalar multiplied by area; the total surface area having a certain combination of habitat conditions, multiplied by the

composite probability of use by fish for the combination of conditions (de Kerckhove et al 2008, Loughlin and Clarke 2014).

Withdrawal: a loss of habitat units or other equivalency that represents the functions that were lost (e.g., productivity, fish density, biodiversity, suitable habitat); the value subtracted from a habitat account ledger; a development project's value as lost habitat or as a measure of serious harm, or residual impact.

REFERENCES

Arlidge, W.N.S., J.W.Bull, R.F.E. Addison, M.J. Burgass, D. Gianuca, T.M. Gorham, C. Jacob, N. Shumway, S.P. Sinclair, J.E. M. Watson, C. Wilcox, E.J. Milner-Gulland. . 2018 A global mitigation hierarchy for nature conservation. BioScience 68: 336-347.

UNEP-WCMC [United Nations Environment Programme – World Conservation Monitoring Centre]. 2014. Biodiversity A-Z website: www.biodiversitya-z.org, UNEP-WCMC, Cambridge, UK.

Bull JW, Gordon A, Watson JEM, Maron M (2016) Seeking convergence on the key concepts in 'no net loss' policy. J Appl Ecol. https://doi.org/10.1111/1365-2664.12726

BBOP (Business Biodiversity and Offsets Programme) (2012) Glossary. Business and Biodiversity Offsets Programme, Washington D.C., U.S.A. 2nd updated edition

Cooley D, Olander L (2011) Stacking Ecosystem Services Payments: Risks and Solutions. Nicholas Institute for Environmental Policy Solutions Working Paper NI WP 11-04. 28 pp.

de Kerckhove DT, Smokorowski KE, Randall RG (2008) A primer on fish habitat models. Can Tech Rep Fish Aquat Sci 2817:iv+65p. Available from https://waves-vagues.dfo-mpo.gc.ca/Library/335800.pdf. Accessed 18 October 2021

DFO (Fisheries and Oceans Canada) (2004) Identification of Ecologically and Biologically Significant Areas. DFO Can Sci Advis Sec Ecosystem Status Rep 2004/006:15p. Available from https://waves-vagues.dfo-mpo.gc.ca/Library/314806.pdf. Accessed 18 October 2021

DFO (Fisheries and Oceans Canada). 2019a. Fish and Fish Habitat Protection Policy Statement. Fisheries and Oceans Canada, Ottawa, Ontario. Available from https://waves-vagues.dfo-mpo.gc.ca/Library/40971193.pdf [accessed 4 January 2022].

DFO (Fisheries and Oceans Canada). 2019b. Policy for Applying Measures to Offset Adverse Effects on Fish and Fish Habitat Under the Fisheries Act. Fisheries and Oceans Canada, Fish and Fish Habitat Protection Program, Ottawa, Ontario. Available from https://waves-vagues.dfo-mpo.gc.ca/Library/40939698.pdf [accessed 8 December 2021].

Doran GT (1981) "There's a S.M.A.R.T. way to write management's goals and objectives". Management Rev AMA FORUM. 70(11):35–36

Fisheries Act, RSC 1985, c F-14. Available from http://canlii.ca/t/543j4 [accessed 6 August 2020].

Gillenwater M (2012) What is additionality? Part 3: Implications for stacking and unbundling. In GHG Management Institute, Discussion Paper n. 3/ vers. 03 (Issue 001).

Hunt K, Patrick P, Connell M (2011) Fish habitat banking in Canada: opportunities and challenges. Econ Commer Anal Rep 180:vi+66

IUCN (International Union for the Conservation of Nature) (2016) Appendix 1 to Biodiversity Offsets Policy, WCC-2012-Res-110-EN, Biodiversity offsets and related compensatory approaches In: WCC-2016-Res-059-EN IUCN Policy on Biodiversity Offsets. Available

from https://portals.iucn.org/congress/assembly/motions/print?langua Accessed 18 October 2021

Lausche, B. (2019). Integrated planning. Policy and law tools for biodiversity conservation and climate change. Gland, Switzerland: IUCN. xvi + 120 pp.

Loughlin KG, Clarke KD (2014) A review of methods used to offset residual impacts of development projects on fisheries productivity. DFO Can Sci Advis Sec Res Doc 2013/097:vi+72. Available from https://waves-vagues.dfo-mpo.gc.ca/Library/361284.pdf. Accessed 18 October 2021

Minns CK, Moore JE, Stoneman M, Cudmore-Vokey B (2001) Defensible Methods of Assessing Fish Habitat: Lacustrine Habitats in the Great Lakes Basin – Conceptual Basis and Approach Using a Habitat Suitability Matrix (HSM) Method. Can Manuscr Rep Fish Aquat Sci 2559:viii + 70 p. Available from https://publications.gc.ca/collections/collection_2007/dfo-mpo/Fs97-4-2559E.pdf Accessed 18 October 2021

Prime L, Lake C, Matos L, Morrison B, Morton E, Portiss R, Snodgrass WJ, Valere BG (2013) The Toronto Waterfront Aquatic Habitat Restorations Strategy: A unique collaborative approach to streamlining approvals and restoring aquatic habitat. Plan Canada. 24–29.

Smokorowski KE, Bradford MJ, Clarke KD, et al (2015) Assessing the Effectiveness of Habitat Offset Activities in Canada: Monitoring Design and Metrics. Can Tech Rep Fish Aquat Sci 3132:vi+48

TRCA (Toronto and Region Conservation Authority) (2003) Toronto Waterfront Aquatic Habitat Restoration Strategy (TWAHRS). Electronic Publication, Available from https://trca.ca/conservation/aquatic-habitat-toronto/strategy. Accessed August 2018

zu Ermgassen SOSE, Baker J, Griffiths RA, Strange N, Struebig MJ, Bull JW (2019) The ecological outcomes of biodiversity offsets under "no net loss" policies: A global review. Conserv. Lett. 12. https://doi.org/10.1111/conl.12664
